# Supplementary figures and images for: A hyperspectral method to assay the microphysiological fates of nanomaterials in histological samples
Source: eLife. 2016 Aug 18;5:e16352. doi: 10.7554/eLife.16352 (PMC5042654; doi:10.7554/eLife.16352)

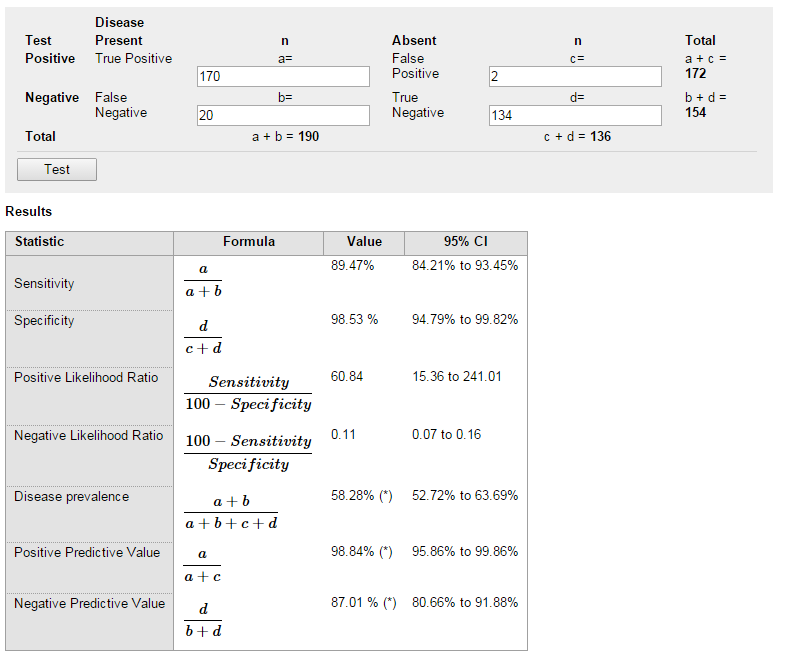

Supplement: Figure 2—source data 1. — DOI: http://dx.doi.org/10.7554/eLife.16352.009 [file elife-16352-fig2-data1.zip › Sensitivity Specificity Raw Data Files/Statistics calculator comparison to ground truth (Relevant for Figure 2).png]

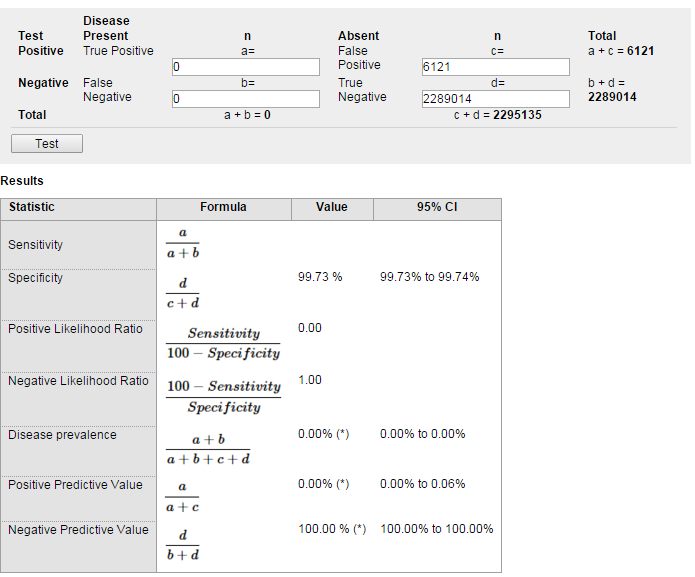

Supplement: Figure 2—source data 1. — DOI: http://dx.doi.org/10.7554/eLife.16352.009 [file elife-16352-fig2-data1.zip › Sensitivity Specificity Raw Data Files/Statistics calculator specificity (Relevant for Figure 2).png]

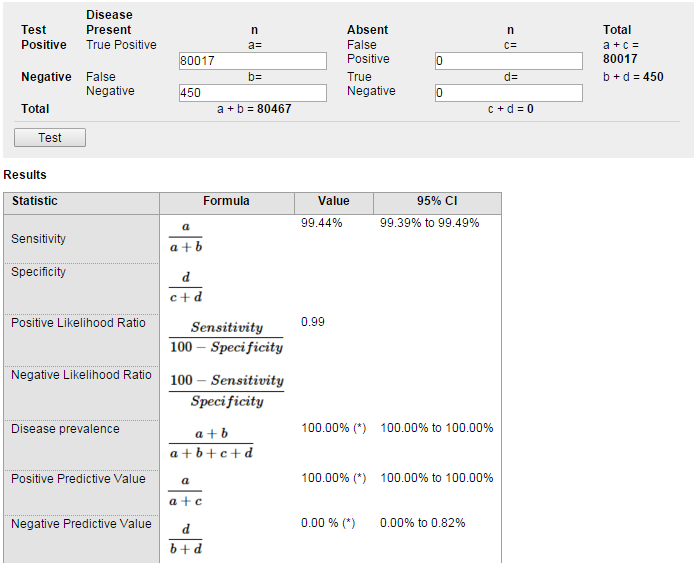

Supplement: Figure 2—source data 1. — DOI: http://dx.doi.org/10.7554/eLife.16352.009 [file elife-16352-fig2-data1.zip › Sensitivity Specificity Raw Data Files/Statitstics calculator sensitivity (Relevant for Figure 2).png]
